# Mitochondria-Mediated Protein Regulation Mechanism of Polymorphs-Dependent Inhibition of Nanoselenium on Cancer Cells

**DOI:** 10.1038/srep31427

**Published:** 2016-08-12

**Authors:** Ge Wang, Yuming Guo, Gai Yang, Lin Yang, Xiaoming Ma, Kui Wang, Lin Zhu, Jiaojiao Sun, Xiaobing Wang, Hua Zhang

**Affiliations:** 1 Collaborative Innovation Center of Henan Province for Green Manufacturing of Fine Chemicals, Key Laboratory of Green Chemical Media and Reactions, Ministry of Education, School of Chemistry and Chemical Engineering, Henan Normal University, Xinxiang, Henan 453007, P. R. China; 2 School of Basic Medical Sciences, Xinxiang Medical University, Xinxiang, Henan 453003, P. R. China; 3 Henan Key Laboratory of Green Chemical Media and Reactions, Henan Normal University, Xinxiang, Henan 453007, P. R. China

## Abstract

The present study was (i) to prepare two types of selenium nanoparticles, namely an amorphous form of selenium quantum dots (A-SeQDs) and a crystalline form of selenium quantum dots (C-SeQDs); and (ii) to investigate the nano-bio interactions of A-SeQDs and C-SeQDs in MCF-7, HepG2, HeLa, NIH/3T3, L929 cells and BRL-3A cells. It was found that A-SeQDs could induce the mitochondria-mediated apoptosis, necrosis and death of cells, while C-SeQDs had much weaker effects. This polymorphs-dependent anti-proliferative activity of nano-selenium was scarcely reported. Further investigation demonstrated that A-SeQDs could differentially regulate 61 proteins and several pathways related to stress response, protein synthesis, cell migration and cell cycle, including “p38 MAPK Signaling”, “p53 Signaling”, “14-3-3-mediated Signaling”, “p70S6K Signaling” and “Protein Ubiquitination Pathway”. This was the first report to demonstrate the involvement of protein synthesis and post-translational modification pathways in the anti-proliferative activity associated with NMs. Compared with previously fragmentary studies, this study use a nanomics approach combining bioinformatics and proteomics to systematically investigate the nano-bio interactions of selenium nanoparticles in cancer cells.

Over the past several decades, a large number of nanomaterials (NMs) with metallic, non-metallic, and organic natures, are widely used in therapeutics, nanoimaging, and biosensing^1^,^2^,^3^,^4^,^5^,^6^. It has been shown that DNA conjugated gold nanoparticle networks have a potent antiviral ability and protect host cells from attack of respiratory syncytial virus^7^. Some non-metallic inorganic nano-species such as silica and carbon have been widely applied in drug delivery, biological imaging, and photothermal therapy^8^,^9^,^10^,^11^,^12^. Polyaniline and polypyrrole organic nanoparticles can be used as near-infrared light-absorbing agents for photothermal therapy of cancer^13^,^14^. In addition, NMs can been emitted into environment and food chains because of the combustion of fossil fuels and industrial production, raising a health concern about the potential toxicities of NMs^15^,^16^,^17^,^18^,^19^. Therefore, it is critical to understand the molecular mechanisms underlying the biomedical activities or the health risks of non-metallic inorganic and organic NMs.

Nanomics, aiming to study the interactions of nanomaterials with genes, proteins and other biomolecules in biological systems by integrating the proteomics, genomics, and metabonomics with bioinformatics, is a more and more attractive research field. Through protein classification and annotation, enrichment analysis and correlation analysis from omics and bioinformatics analysis, the data can be scientifically interpreted and the biological significance can be pinpointed^20^. In this regard, several studies about the nano-bio interactions based on omics techniques have been reported^21^,^22^,^23^,^24^,^25^,^26^. In addition, fascinating polymorphs-dependent bioactivity of NMs has been reported recently, such as copper sulphide nanoparticles^27^. However, the underlying mechanisms still remain unexplored. Meanwhile, selenium compounds attracted more and more attention in the past decade due to their high bioavailability and antioxidant activities, low toxicity, and novel therapeutic properties^28^,^29^. But the polymorphs-dependent anti-proliferative activity of nano-selenium was scarcely reported.

In the current study, two nanoparticles were firstly prepared, namely amorphous selenium quantum dots (A-SeQDs) and crystalline selenium quantum dots (C-SeQDs). The subsequent anti-proliferative effects on cancer cells were investigated, demonstrating that SeQDs exhibited anti-proliferative effects on cancer cells in a polymorphs-dependent manner. A-SeQDs rather than C-SeQDs could specifically inhibit the proliferation of cancer cells. Finally, we explored the mechanisms underlying the polymorphs-dependent anti-proliferative activity of nanoscaled selenium. The results suggested that A-SeQDs was effective in inducing mitochondria-mediated apoptosis and necrosis (Fig. 1). It was speculated that A-SeQDs could enter directly mitochondria of cancer cells and regulate apoptosis-associated pathways including “p38 MAPK Signaling”, “p53 Signaling” and “14-3-3-mediated Signaling”. Moreover, the changes of mitochondria could further affect the endoplasmic reticulum (ER) through the interaction of mitochondria with ER and regulate the pathways involved in protein synthesis and post-translational modification including “p70S6K Signaling” and “Protein Ubiquitination Pathway”. This was the first report to demonstrate the involvement of protein synthesis and post-translational modification pathways in the anti-proliferative activity associated with NMs.

## Results and Discussion

### Preparation and characterization of SeQDs

**Figure 2:**
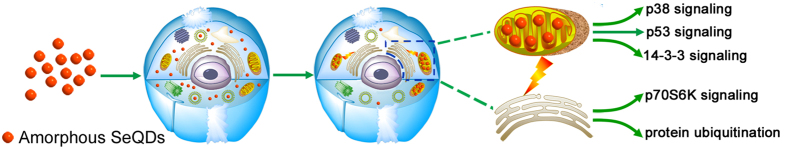
Proposed mechanisms of anti-proliferative effects of A-SeQDs on cancer cells.

In this study, two polymorphs of SeQDs were prepared through self-redox decomposition of selenosulfate precursor in the presence of bovine serum albumin. From the X-ray diffraction (XRD) pattern (lower curve in Fig. 2a), the sample prepared under 20 °C did not exhibit any obvious diffraction peaks, indicating the successful formation of amorphous phase (A- SeQDs). The energy-dispersive X-ray (EDX) spectrum (inset of Fig. 2a) indicated that the composition of the sample is selenium. Compared with A-SeQDs, the XRD pattern of the sample prepared under 80 °C (upper curve in Fig. 2a) presented the sharp diffraction peaks corresponding to the (100), (101), (110), (102), (111), (201), (112), (202) and (210) planes of the hexagonal Se (JCPDF 06-0362), respectively, indicating the formation of crystalline phase (C-SeQDs). These results demonstrated that two SeQDs with different polymorphs could be prepared through the facile adjustment of the preparation conditions.

The high resolution-transmission electron microscopy (HRTEM) observations indicated the different sizes and polymorphs of the different SeQDs. From the results shown in Fig. 2c–e, the two samples were both the well-dispersed QDs with the average diameters of 2.25 ± 0.19 nm for A-SeQDs (Fig. 2c) and 4.10 ± 0.04 nm for C-SeQDs (Fig. 2e). In addition, the existence of a diffused halo ring rather than any detectable rings or spots in the selected-area electron diffraction (SAED) pattern of A-SeQDs (inset of Fig. 2c) further revealed the formation of the amorphous product. In comparison, the SAED pattern of C-SeQDs exhibits the obvious diffraction spots (inset of Fig. 2e), further confirming the formation of the crystalline product.

**Figure 3:**
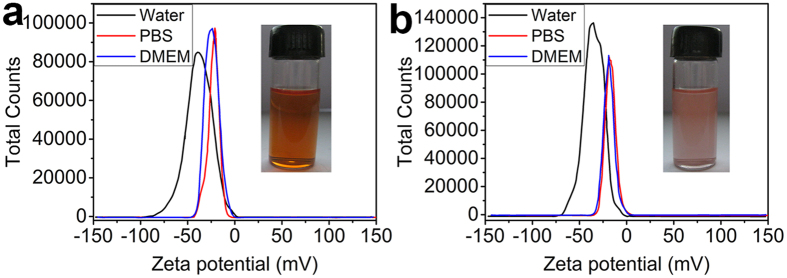
(**a**) ξ-potentials of A-SeQDs in different media. Inset: digital photograph of the A-SeQDs dispersion in PBS. (**b**) ξ-potentials of C-SeQDs in different media. Inset: digital photograph of the C-SeQDs dispersion in PBS.

Results from the ξ-potential measurement revealed that both SeQDs samples had the good stability under the physiological conditions. The ξ-potentials of the samples in H_2_O, phosphate buffer solution (PBS), and Dulbecco’s modified Eagle medium (DMEM) were determined to be −38.5, −21.3, and −23.8 mV for A-SeQDs (Fig. 3a) and −35.5, −17.1, and −18.3 mV for C-SeQDs (Fig. 3b), respectively, proving that two SeQDs possessed good stability and carried net negative charges under the physiological conditions. The formation of the stable and clear dispersions of two SeQDs in PBS further confirmed the stability of SeQDs under the physiological conditions (inset of Fig. 3a,b).

**Figure 4:**
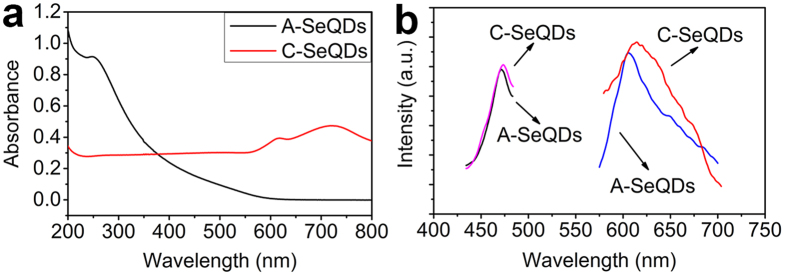
(**a**) UV-Vis absorption spectra of SeQDs. (**b**) PL and excitation spectra of SeQDs. right : excitation spectra of SeQDs ; left : PL spectra of SeQDs

Optical properties of SeQDs were determined. The UV-Vis spectra showed that A-SeQDs exhibited stronger absorbance between 200~377 nm and weaker absorbance between 378~800 nm than C-SeQDs (Fig. 4a). The solid photoluminescence (PL) determination revealed that SeQDs had the good PL properties (right in Fig. 4b). Results demonstrated that QDs exhibited the emission peak centered at 605 nm for A-SeQDs and 614 nm for C-SeQDs when excited with light at a wavelength of 475 nm. In order to further test the PL properties of SeQDs, the excitation spectra were measured. The emission wavelength was set in 605 nm for A-SeQDs and 614 nm for C-SeQDs. From the results (left in Fig. 4b), the absorption peaked at 476 nm for A-SeQDs and 478 nm for C-SeQDs. These results were in agreement with the above-mentioned emission result, further confirming the PL property of the SeQDs.

### Detection of cytobiology activity and internalization of two SeQDs

**Figure 5:**
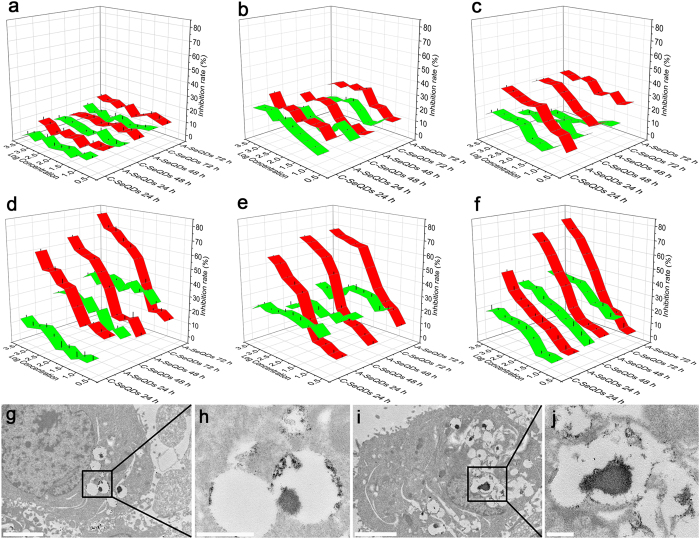
The anti-proliferative effects of A-SeQDs and C-SeQDs on different cells. (**a**) NIH 3T3 cells; (**b**) L929 cells; (**c**) BRL-3A cells; (**d**) Hep G2 cells; (**e**) MCF7 cells; (**f**) HeLa cells. Red bands show the results of A-SeQDs, green bands show the results of C-SeQDs. TEM images of Hep G2 cells treated with (**g**, **h**) A-SeQDs and (**I, j**) C-SeQDs. Panels h and j show the higher-magnification images of the rectangle framed area in panels g and i. Dark spots show the SeQDs. Scale bar in e, g: 2 μm; scale bar in (**f, h**): 500 nm.

The anti-proliferative effects of two SeQDs forms on various cells were determined using thiazolyl blue tetrazolium bromide (MTT) assay. The results indicated that SeQDs exhibited the polymorphs-dependent anti-proliferative effects on various cancer cells. In Fig. 5, the red bands represent the anti-proliferative effects of A-SeQDs on different cells with different treatment times, while the green bands represent those of C-SeQDs. IC_50_ values of A-SeQDs in Hep G2 (Fig. 5d), MCF-7 (Fig. 5e), and HeLa cells (Fig. 5f) are 0.0757 mM, 0.0953 mM, and 0.2754 mM, respectively. However, the IC_50_ values in normal cells including NIH 3T3, L929, and BRL-3A cells were undetectable (Fig. 5a–c), suggesting that A-SeQDs selectively inhibited the proliferation of cancer cells. Furthermore, with the increase of doses (5 nM to 2.56 mM) and treatment time (24 to 72 h), the anti-proliferative effects of A-SeQDs significantly increased in a dose-dependent and time-dependent manner. In contrast, C-SeQDs just slightly inhibited the proliferation of all the test cell lines and the IC_50_ values were undetectable, suggesting that the anti-proliferative activity of SeQDs is polymorphs-dependent.

**Figure 6:**
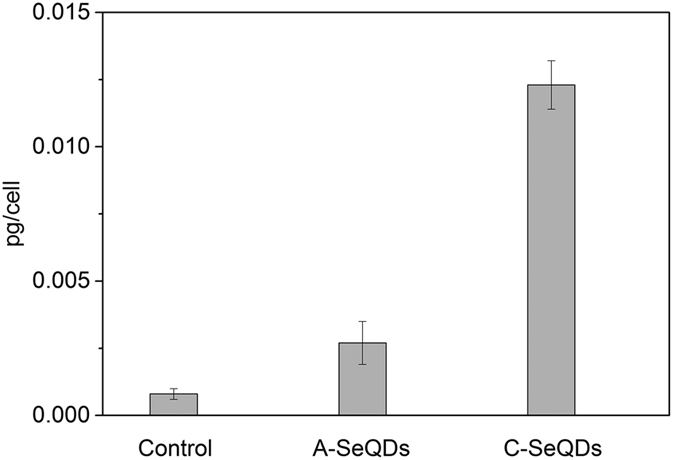
Intracellular SeQDs concentrations in Hep G2 cells.

Given the important functions of internalization for the anti-proliferative effects of NMs, the internalization of two SeQDs were determined by transmission electron microscopy (TEM) observations using Hep G2 cells as an example. Results showed that A-SeQDs (dark spots in Fig. 5g,h) and C-SeQDs (dark spots in Fig. 5I,j) could be both effectively internalized into Hep G2 cells *via* vacuole-like vesicles (or membrane-bound vesicles), and tended to form clusters. Furthermore, the cellular uptake efficiencies of SeQDs were evaluated by ICP-MS analysis. From the results shown in Fig. 6, the intracelleular concentrations of SeQDs in Hep G2 cells are significant different. The intracelleular concentration of C-SeQDs (0.0123 pg/cell) is about 3.5 times higher than that of the A-SeQDs (0.0027 pg/cell) and 15.4 times higher than that of the control (0.0008 pg/cell). It is surprising that the higher intracelleular concentration of C-SeQDs than A-SeQDs does not lead to the stronger anti-proliferative effect on cancer cells, suggesting the intersample dose-independent anti-proliferative effects.

### Cell Cycle and apoptosis determination by flow cytometric analysis

**Figure 7:**
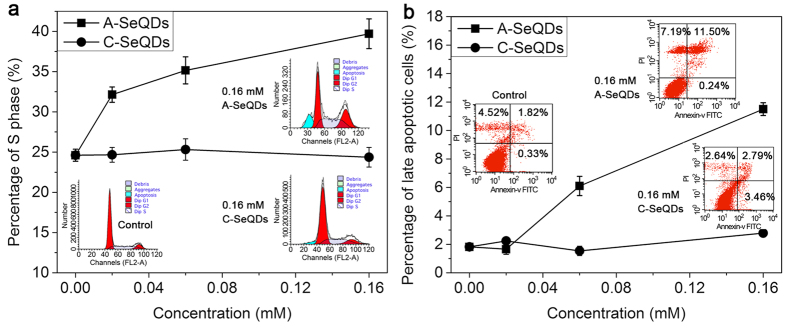
(**a**) Effects of SeQDs on the S phase of the Hep G2 cells after treated for 72 h. Inset: Cell cycle distribution of the control group and the cells exposed to 0.16 mM SeQDs. Cyan peak: apoptotic and necrotic cells. (**b**) Apoptosis evaluation of Hep G2 cells treated with SeQDs for 72 h. Inset: Flow cytometry profiles of the control group and the cells exposed to 0.16 mM SeQDs. The upper left, upper right, bottom left, and bottom right quadrants of each panel represent the necrotic, late apoptotic, early apoptotic, and viable cells, respectively.

To determine the possible mechanisms of the polymorphs-dependent anti-proliferative activity of SeQDs, effects of two SeQDs on the cell cycle distribution were analyzed by flow cytometric analysis. As shown in Fig. 7a, Hep G2 cells were easier to be arrested in S phase after A-SeQDs treatment. When treated by 0.16 mM of A-SeQDs for 72 h, the percentage of the S phase increased from 24.61% of the control group to 39.70%. However, the percentage of the S phase treated by C-SeQDs was 24.36%, almost as same as the control group. Moreover, the cyan peak of the apoptotic and necrotic cells of the A-SeQDs treatment group was significantly higher than that of the C-SeQDs treatment group (Inset of Fig. 7a), most likely attributable to the stronger inhibition effects of A-SeQDs on mitosis and proliferation of cancer cells. These results were well in agreement with the above anti-proliferation results.

To distinguish the effects of two SeQDs on apoptosis and necrosis of cancer cells, the percentages of the dead, viable, apoptotic and necrotic cells of Hep G2 cells were determined by Annexin V-fluorescein isothiocyanate (FITC)/propidium iodide (PI) double-staining flow cytometric analysis. Results showed that A-SeQDs rather than C-SeQDs could significantly induce the late apoptosis (upper right quadrant in inset of Fig. 7b) of cancer cells. After treated by 0.16 mM of A-SeQDs for 72 h, the percentage of the late apoptotic cells significantly increased to 11.5%, much higher than those of the control group (1.82%) and the C-SeQDs treatment group (2.79%) (insert of Fig. 7b). These results suggested that the different anti-proliferative effects of A-SeQDs and C-SeQDs on cancer cells were polymorphs-dependent at late apoptosis stage.

### Co-localization with the mitochondria by confocal laser scan microscopy

**Figure 8:**
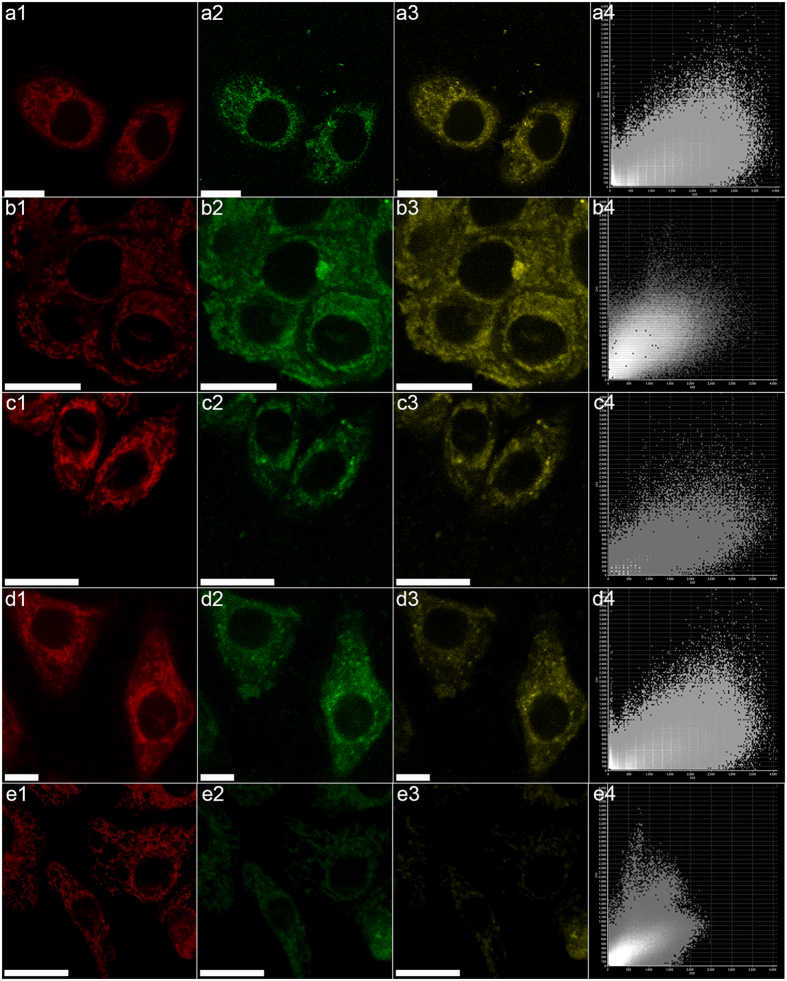
CLSM images of the different cells incubated with different samples. (**a**) Hep G2 cells incubated with A-SeQDs; (**b**) MCF-7 cells incubated with A-SeQDs; (**c**) HeLa cells incubated with A-SeQDs; (**d**) BRL-3A cells incubated with A-SeQDs; (**e**) Hep G2 cells incubated with C-SeQDs; (1) Mitochondria Tracker; (2) A-SeQDs; (3) Dark-field image of Mitochondria Tracker + A-SeQDs; (4) Intensity correlation plot of Mitochondria Tracker and A-SeQDs. Scale bar: 20 μm.

Considering the significant apoptosis-inducing effect of A-SeQDs on cancer cells and the important roles of mitochondria in the regulation of apoptosis^30^,^31^, the mitochondria of the model cells exposed to A-SeQDs were visualized by confocal laser scan microscopy (CLSM) to investigate whether A-SeQDs could co-localize with the mitochondria. Meanwhile the co-localization coefficient was evaluated by Pearson’s correlation factor^32^. From the results (Fig. 8), the fluorescence of A-SeQDs(Fig. 8a2) and the red fluorescence of the Mitochondrial Tracker (Fig. 8a1) overlapped well each other. This clearly indicates that A-SeQDs could co-localize with the mitochondria of Hep G2 cells. The Pearson’s correlation factor was determined as 0.80 (Fig. 8a4), indicating the preferential accumulation of A-SeQDs mainly in the mitochondria of Hep G2 cells^33^. From Fig. 8b–d, A-SeQDs can also preferentially accumulate in the mitochondria of MCF-7, HeLa, and BRL-3A cells. The Pearson’s correlation factors were determined as 0.77, 0.78, and 0.83, respectively. From the results shown in Figure S1, similar to A-SeQDs, C-SeQDs can also preferentially accumulate in the mitochondria of the model cells. However, from Fig. 8e and Figures S2 and S3, the fluorescence of A-SeQDs and C-SeQDs and the red fluorescence of the Lyso-Tracker just slightly overlapped each other. The Pearson’s correlation factor for Hep G2 cells was determined as 0.13. These results suggest that SeQDs could not preferentially accumulate in the lysosome of model cells. Furthermore, Δψm of Hep G2 cells exposed to SeQDs were measured. The results show that SeQDs could lead to the dose-dependent loss of Δψm (see Supplementary Figure S4). More importantly, the effect of A-SeQDs on the loss of Δψm was higher that of C-SeQDs, correlated well with their apoptosis-inducing effects. This revealed that A-SeQDs could efficiently breakdown the mitochondrial membrane of cancer cells and led to the apoptosis. Therefore the mitochondria-induced endogenous apoptosis should play the important role in the inhibition of the cancer cells proliferation by A-SeQDs.

Based on the above results, A-SeQDs could inhibit the proliferation of cancer cells through the cellular uptake and mainly localization to mitochondria, breakdown of the mitochondrial membrane, depletion of the mitochondria potential, and induction of the apoptosis and the cell cycle arrest in S phase.

### Proteomics analysis based on bioinformatics

**Figure 9:**
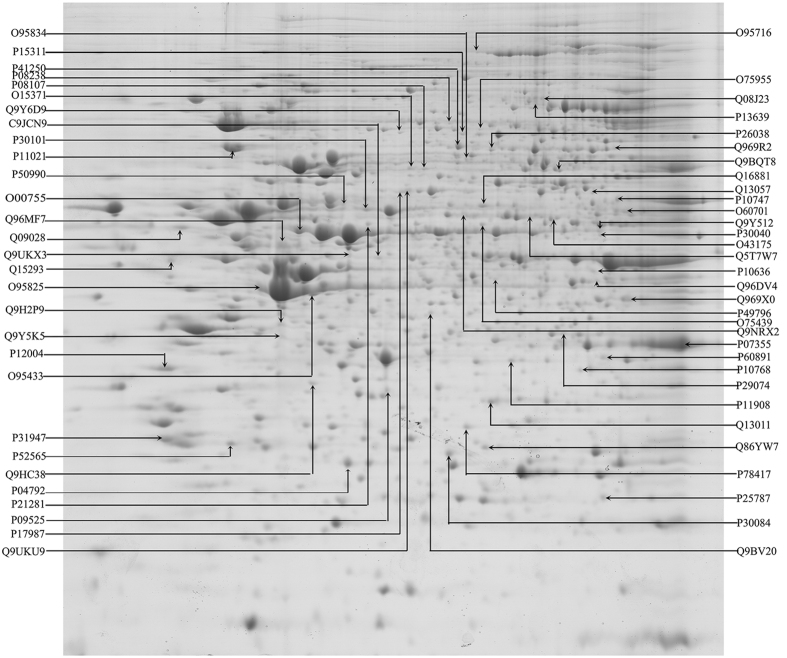
Maps of coomassie blue-stained 2DE gel from Hep G2 whole cell lysate focused on a non-linear pH 3-10 IPG strip. The significantly differentially expressed protein spots were identified. The Uniprot accession number of each protein is shown.

To systematically and deeply study the molecular mechanisms of the polymorphs-dependent anti-proliferative effects of SeQDs on cancer cells, proteins regulated by SeQDs were detected using nanomics. The representative 2D gel images for whole cell proteins extracted from Hep G2 cells were shown in Fig. 9. The proteomic maps of the control and the treated cells were compared using ImageMaster 2D Platinum 7.0 software to identify the protein spot variations. After treatment, significantly differentially expressed protein spots (p < 0.05) with 1.5-fold change in intensity were scored. Based on the differentially expressed proteins for A-SeQDs treatment, 61 proteins including 29 up-regulated and 32 down-regulated proteins could be identified. Tables 1 and S1 (see Supplementary Information) presented the identified proteins.

**Figure 10:**
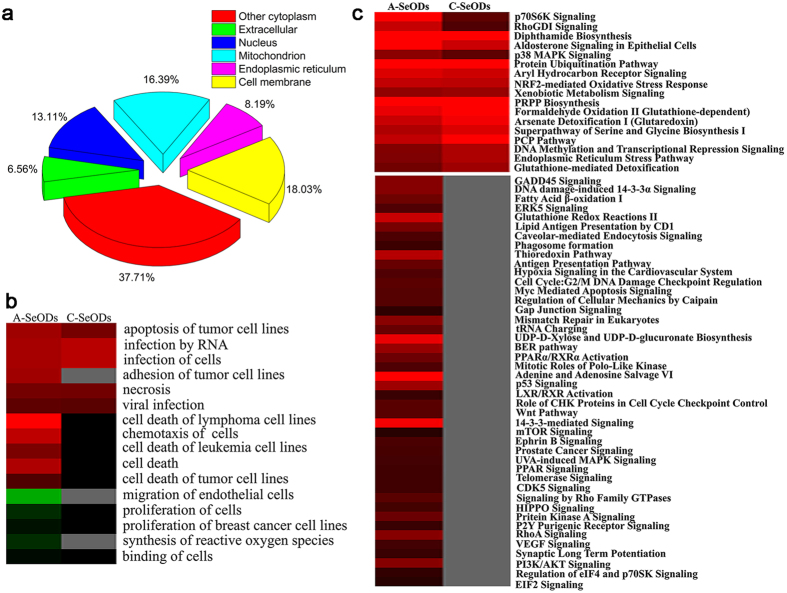
(**a**) The cellular distributions of the identified proteins. (**b**) The clustered biological function patterns of the identified proteins. The red color represents the promotion effect on the related process. The green color represents the inhibition effect on the related process. The grey and black colors represent the nonsignificant influence. (**c**) The significantly enriched canonical pathways of the identified proteins. The red color represents the significant effects on the canonical biological pathways. The brighter the red color is, the stronger the effect is. The grey and black colors represent the nonsignificant influence.

The identified proteins are categorized based on their cellular locations, namely cytoplasm (37.71%), cell membrane (18.03%), mitochondrion (16.39%), nucleus (13.11%), endoplasmic reticulum (ER, 8.19%), and extracellular matrix (6.56%), respectively (Fig. 10a). Furthermore, in order to better understand the biological relevance of the changes in protein expression in response to SeQDs, the proteins are classified into 7 groups based on their functions, including stress response, signaling pathway and transduction, protein biosynthesis and metabolism, cell cycle, cell adhesion and migration, metabolism, and oxidation-reduction process.

To systematically and comprehensively study the anti-proliferative mechanisms of A-SeQDs and C-SeQDs, bioinformatics including Ingenuity Pathway Analysis (IPA) and hierarchical clustering analysis were used to compare the protein expression profiles between the samples. The studies indicated that different SeQDs exhibited discriminatively prominent effects on biological functions and canonical pathways of Hep G2 cells. The biological function patterns of the identified proteins were clustered and the results were shown in Fig. 10b. Firstly, A-SeQDs induced apoptosis, necrosis and death of the tumor cells, while C-SeQDs had much weak effects on these cellular processes, which were consistent with the above results. Secondly, it was logical that the functional enrichment and clustering analysis was to mainly focus on death and survival of the tumor cells.

To further clarify the important signaling pathways related to the anti-proliferative activities of SeQDs in Hep G2 cells, pathway analysis was performed to connect the differentially expressed proteins with canonical biological pathways by IPA software. A Benjamini-Hochberg corrected Fischer’s exact test was utilized to calculate the p-value associated with a canonical pathway (Fig. 10c). Results showed that A-SeQDs exhibited more pronounced and global impact on various canonical pathways than C-SeQDs, especially the pathways in relation to cell survival such as the apoptosis pathways “p53 Pathway”, “p38 MAPK Signaling” and “14-3-3 mediated Signaling”^34^, and protein synthesis such as “PI3K/AKT-mTOR/p70S6K Signaling” and “Protein Ubiquitination Pathway”^35^,^36^. Although previous studies indicated that the anti-proliferative activities of selenium-containing compounds can be attributed to the Akt/Mdm2/AR controlled apoptosis or induction of S phase arrest^37^,^38^, our results reveal that cooperation of multiple pathways generates functional difference between different Se samples.

**Figure 11:**
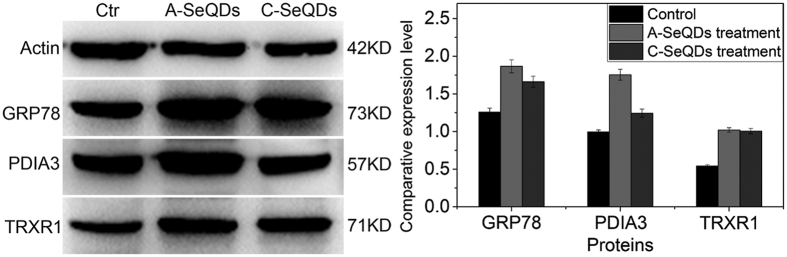
(**a**) Effects of SeQDs on expression levels of β-actin, GRP78, PDIA3, and TRXR1 analyzed by Western blotting. The full-length blots of β-actin, GRP78, PDIA3, and TRXR1 are presented in Supplementary Figure S5, respectively. (**b**) The relative intensity of bands was calculated with corresponding actin and plotted. Each bar represents the mean ± SD. (n = 3).

Subsequently, the proteins with the important roles in multiple pathways are summarized and discussed. As suggested by IPA results (see Supplementary Table S2), some differentially expressed proteins may play multiple roles in mediating multiple pathways. For example, the level of 14-3-3 protein in Hep G2 cells exposed to A-SeQDs showed significant down-regulation over 7-fold (no significant change exposure to C-SeQDs) in comparison with control group. As a family member of multifunctional phosphoserine binding molecules, 14-3-3 protein serves as an effector of survival signaling^39^. This study demonstrates that when treated by A-SeQDs, down-regulation of 14-3-3 protein could exert direct or indirect influence on differential changes of multiple pathways, especially “p70S6K Signaling”, “p53 Pathway”, “14-3-3 mediated Signaling”, “DNA damage-induced 14-3-3σ Signaling”, and “Cell Cycle: G2/M DNA Damage Checkpoint Regulation” (see Supplementary Table S1). It means that A-SeQDs might exhibit the anti-proliferative activity through the regulation of the 14-3-3 expression and the related multiple pathways, not just limited to the previous reports. In addition, thioredoxin reductase 1 (TRXR1) showed approximately 3-fold up-regulation upon exposed to A-SeQDs, which was confirmed by western blotting analysis (Fig. 11, see Supplementary Figure S5 for full-length blots online). As a key element of the thioredoxin system, this protein applies to the detoxification of reactive oxygen metabolites and the signaling processes^23^. The overexpression of TrxR implied the disorder of the intracellular redox homeostasis, which might induce the significant apoptosis of the target cells.

Our analysis showed that A-SeQDs exhibited significant influence on heat shock proteins (HSPs) (Table 1). The levels of HSPs family members (HSP90, HSP70, and HSP27), except for the heat shock factor-binding protein (HSPBL), were significantly down-regulated upon exposure to A-SeQDs rather than to C-SeQDs. However, HSPBL was significantly up-regulated upon exposure to A-SeQDs rather than C-SeQDs. It is well-known that HSPs are one of the most conserved cytoprotective warriors against apoptosis and oxidative stress^40^,^41^. The down-regulations of HSPs induced by A-SeQDs in Hep G2 cell indicated the dysfunction of protection, contributing to the anti-proliferative effects. It was worthy to notice that the changes of HSPs expression in comparison with the control group were related to the changes of multiple pathways especially “Protein Ubiquitination Pathway” and “p38 MAPK Signaling” (see Supplementary Table S2). It could be therefore concluded that HSPs might serve as an indicator of potential anti-proliferation targets or effector of A-SeNPs.

Additionally, ER stress associated proteins 78 kDa glucose-regulated protein (GRP78) and ER resident protein 29 (ERP29) were significantly up-regulated in both A-SeQDs and C-SeQDs treated groups (Table 1). Meanwhile, protein disulfide-isomerase A3 (PDIA3), namely ERP57, showed significant upregulation after A-SeQDs treatment. These results agree well with western blotting analysis results (Fig. 11, see Supplementary Figure S5 for full-length blots online). The overexpression of these proteins could be regarded as the indicator of ER stress response^42^. As reported, over-expression of ERP29 was inversely correlated with tumor progression due to induction of apoptosis and inhibition of cell cycle in a variety of cancer cells^43^. When ER stress happens, ER chaperon (GRP78) dissociates from the ER transmembrane proteins, leading to the activation of transmembrane proteins. Therefore, high-expression of ER-associated proteins can be considered as the self-protective mechanism of HepG2 cell against SeQDs. However, recent findings uncovered that the junction between the ER and the mitochondria played a crucial role in cell death regulation^44^. Under ER stress condition, mitochondria-associated membrane induces apoptosis *via* the ER–mitochondria interaction and further the mitochondria dysfunction^44^. Equivalently differential ER stress response to two polymorphs of SeQDs would be helpful to understand equivalently loss of Δψm (see Supplementary Figure. S1).

## Conclusion

This research was to study the nanomics of two polymorphs of SeQDs including A-SeQDs in cancer cells. It was the first time to demonstrate the anti-proliferative activity of SeQDs was polymorphs-dependent with A-SeQDs being much more effective than C-SeQDs in cancer cells. This was most likely A-SeQDs had more effective influence on breaking the mitochondrial membrane, depleting the mitochondria potential, inducing the apoptosis and the cell cycle arrest in S phase. Nanomics approach was used to study the underlying mechanisms. Results showed that A-SeQDs could alter 61 proteins functionally related to metabolism, protein synthesis and post-translational modification, and involved in various signaling pathways, such as “p53 Pathway”, “p38 MAPK Signaling”, “14-3-3 mediated Signaling”, “p70S6K Signaling”, and “Protein Ubiquitination Pathway”. The protein synthesis and post-translational modification pathways including “p70S6K Signaling” and “Protein Ubiquitination Pathway” were first reported to be involved in the anti-proliferative effects of NMs. These findings propose a molecular basis for further unraveling the mechanisms of the anti-proliferative effects of NMs on cancer cells. The idea employing nanomics as a tool to study the interaction between a nanoparticle and cancer cells is powerful.

## Methods

### Preparation of SeQDs

For the preparation of A-SeQDs, black selenium powder was added into aqueous solution of sodium sulfite (50 mM) at 95 °C. Then BSA (70 mg) was added into the reaction system and the pH was adjusted to 6.0. The reaction system instantly changed to red color. Subsequently, the reaction system was incubated at 20 °C for 12 h. Finally, the dispersion was centrifuged, washed, and freeze-dried. The preparation of C-SeQDs was performed under the similar conditions to those used in the preparation of A-SeQDs, except for the elevated incubation temperature (80 °C) and time (24 h).

### Characterization of SeQDs

The size and morphology of the SeQDs were characterized by HR-TEM (JEOL JEM-2100) with the acceleration voltage of 200 kV. The crystal phases were determined by XRD using a D8ADVANCE X-ray diffractometer (Bruker axs Com.) with graphite monochromatized Cu Kα radiation (λ = 0.15406 nm) in the 2θ range of 20–80°. EDX spectrum was recorded on a GENESIS system (EDAX Inc.) attached to the JEM-2100 microscope. The photoluminescence (PL) measurements were carried out on a HITACHI FP-6500 spectrophotometer. ζ potential was measured on a Nano-ZS instrument in triplicate in H_2_O, PBS, or DMEM.

### Cell lines and cell culture

The cell lines used in this study were all purchased from American Type Culture Collection (ATCC, Manassas, VA), including MCF-7 breast adenocarcinoma cells, Hep G2 human heptoblastoma cells, HeLa human cervical carcinoma cells, and NIH/3T3 mouse fibroblast cells. They were cultured in RPMI-1640, DMEM, or EMEM medium supplemented with 10% heat-inactivated FBS, penicillin (100 units/mL), Streptomycin (100 μg/mL), amphotericin B (0.25 μg/mL) at 37 °C in a humidified incubator at fully humidified atmosphere at 37 °C. 5% CO_2_ and 95% room air.

### Anti-proliferative effects evaluation

The anti-proliferative effects of SeQDs were determined by MTT colorimetric assay. Briefly, culture media (100 μL) containing test cells with initial cell density of 2.5 × 10^4 ^ cells/mL were seeded separately in the wells of sterile 96-well microplates and incubated for 24 h. Then, SeQDs dispersions in culture media with different concentrations were added and incubated for 24 h, 48 h, and 72 h, respectively. The treatment of cells with culture media rather than SeQDs was prepared as the control. After treatment, MTT was added to each well and incubated for 5 h. Then the supernatant was removed and DMSO were added to dissolve the dark blue crystals completely. The absorbance of the solution in each well at the wavelength 570 nm was measured by a MK3 microplate reader (Thermo Fisher Scientific Inc.). The anti-proliferative effects of different samples were calculated by the following equation. The data were reported as mean ± standard deviation (SD) based on triplicate measurements. Statistical analysis was performed using one-way ANOVA statistical package in OriginPro 9.0.


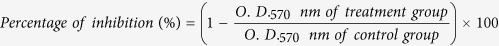


### Detection of apoptosis by flow cytometric analysis

Apoptotic and necrotic cell levels were determined by flow cytometric analysis after double staining with Annexin V-FITC and PI using an assay kit. After incubated with SeQDs for 72 h, the medium was removed and the cells were washed twice with ice-cold PBS. After stained, the cells were collected, washed with ice-cold PBS, and suspended in binding buffer. The cells were analyzed by a FACSCalibur flow cytometer (Becton Dickinson & Co., NJ) and the percentages of apoptotic and necrotic cells were quantified.

### Cell cycle analysis

Well-cultivated Hep G2 cells were treated with SeQDs (0.02, 0.06, and 0.16 mM) for 72 h. Subsequently, cells were collected, washed with ice-cold PBS, and fixed with cold 70% ethanol at 4 °C. Then, cells were washed with ice-cold PBS and resuspended in ice-cold PBS. Finally, cells were incubated with RNase and PI and analyzed by a FACSCalibur flow cytometer.

### CLSM analysis

Hep G2 cells cultured in 35 mm confocal dish with cover glass bottom were stained with Mitochondrial Tracker (1 μg/mL). After washed twice with PBS, cells were cultured in medium containing A-SeQDs for 4 h on a thermo-cell culture FCS2 chamber and analyzed by Carl Zeiss Cell Observer (Jena, Germany).

### Evaluation of loss of Δψm

The mitochondrial membrane potential (Δψm) was determined with the assistance of fluorescent probe JC-1. Hep G2 cells exposed to A-SeQDs (0.02, 0.06, and 0.16 mM) were collected and washed with PBS. Cells were incubated with JC-1 (5 μM), washed and re-suspended in PBS at a density of 1 × 10^6 ^ cells/mL. The fluorescence intensity at the wavelength of 595 nm was measured at an excitation wavelength of 515 nm using a FACSCalibur flow cytometer.

### Inductively Coupled Plasma-Mass Spectrometry (ICP-MS)

Intracellular Se concentration was determined by ICP-MS. Briefly, collected cells were digested with 3 mL of concentrated nitric acid and 1 mL of H_2_O_2 _in an infrared rapid digestion system at 180 °C for 1.5 h. The digested solution was reconstituted to 10 mL with Milli-Q H_2_O and used for ICP-MS analysis (ELAN DRC-e, Perkin–Elmer Sciex).

### TEM observation of cells

Hep G2 cells (5 × 10^4 ^ cells/mL) were treated with SeQDs for 72 h, washed with PBS, and fixed with glutaraldehyde (2.5%). Cells were postfixed in osmium tetroxide, dehydrated in ethanol, and embedded in Epon. Ultrathin sections were stained with uranyl acetate and lead citrate and examined by TEM.

### Protein extraction

Cells were washed with PBS, collected, and re-suspended in lysis buffer containing urea (7 M), thiourea (2 M), CHAPS (4%), dithiothreitol (20 mM), Pharmalyte pH 3–10 (0.5%), protease inhibitor mix, and nuclease mix. Protein concentrations were determined using non-interference protein assay kit (SK3071, Sangon Biotech., China) according to the manufacturer’s instruction.

### Two dimensional gel electrophoresis

For analytical and preparative gels, protein samples (350 μg) were loaded into the rehydrated 24 cm non-linear Immobilized pH gradient (IPG) strip (pH 3–10, nonlinear). The first dimensional isoelectric focusing (IEF) run was carried out using the following conditions: (i) 30 V, 10 h; (ii) 60 V, 3 h; (iii) 100 V, 1.5 h; (iv) 500 V, 1.5 h; (v) 1000 V, 2h; (vi) 1000–10000V, 3h; (vii) 10000 V–140000 Vh. This was followed by the second dimensional SDS-PAGE, performed on 1.0 mm polyacrylamide gels (12.5%) at a constant power of 10 W per gel at 16 °C by using Ettan DALTsix electrophoresis system (Amersham Biosciences). All samples were run in triplicate to ensure reproducibility.

### Protein visualization and image analysis

2 D gels were visualized by staining with Coomassie blue. The stained gels were scanned by an image scanner and all the triplicate gels were then analyzed with ImageMaster 2D Platinum 7.0 software (GE Healthcare).

### MALDI-TOF/MS and MS/MS

The differentially expressed protein dots were cut from the gels to prepare dry extract. Dry extract was re-dissolved in matrix solution (1 mL) containing acyano-4 hydroxycinnamic acid (CHCA, 5 mg/mL) in trifluoroacetic acid (0.1%) and acetonitrile (50%). The extract was spotted onto the MALDI target plate and allowed to dry in air prior to MS analysis using an MALDI-TOF/TOF Mass Spectrometer (Applied Biosystems 4800 Proteomics Analyzer, Framingham). The peptides and proteins were identified by using GPS explorer software Version 3.6 (Applied Biosystems) and MASCOT search engine (Version 2.1; Matrix Science).

### Western blotting

Protein extracted from cell lysates as described above was resolved on SDS-PAGE gel and transferred onto PVDF membrane *via* semidry transfer (BioRad). Membranes were blocked in 3% BSA and washed in Tris-buffered saline with 0.1% Tween. Membranes were incubated with 1:500 primary antibody (anti-β-actin, anti-Grp78, anti-PDIA3, anti-TRXR1, purchased from Abcam) followed by the corresponding secondary antibody with 3 washing steps in between. Protein bands were developed with chemiluminescence kit.

### Bioinformatics analysis

Categorization of protein functions was performed based on Uniprot/TrEMBL database search. Ingenuity Pathway Analysis (IPA, http://www.ingenuity.com) was used to determine the distribution of identified proteins and their participation in molecular networks related to carcinogenesis. Detailed information of the identified proteins including accession number and expression changes were imported to IPA software to perform the analysis based on Ingenuity Knowledge Base. The functions and involved pathways of each protein obtained from IPA analysis was then clustered by Cluster 3.0 and TreeView software.

### Statistical analysis

All the biological experiments were carried out at least in triplicate, and the results were expressed as mean ± SD. Differences between two groups were analyzed by one-way analysis of variance (ANOVA) multiple comparisons.

## Additional Information

**How to cite this article**: Wang, G. *et al*. Mitochondria-Mediated Protein Regulation Mechanism of Polymorphs-Dependent Inhibition of Nanoselenium on Cancer Cells. *Sci. Rep.* **6**, 31427; doi: 10.1038/srep31427 (2016).

## Supplementary Material

Supplementary Information

## Figures and Tables

**Figure 1:**
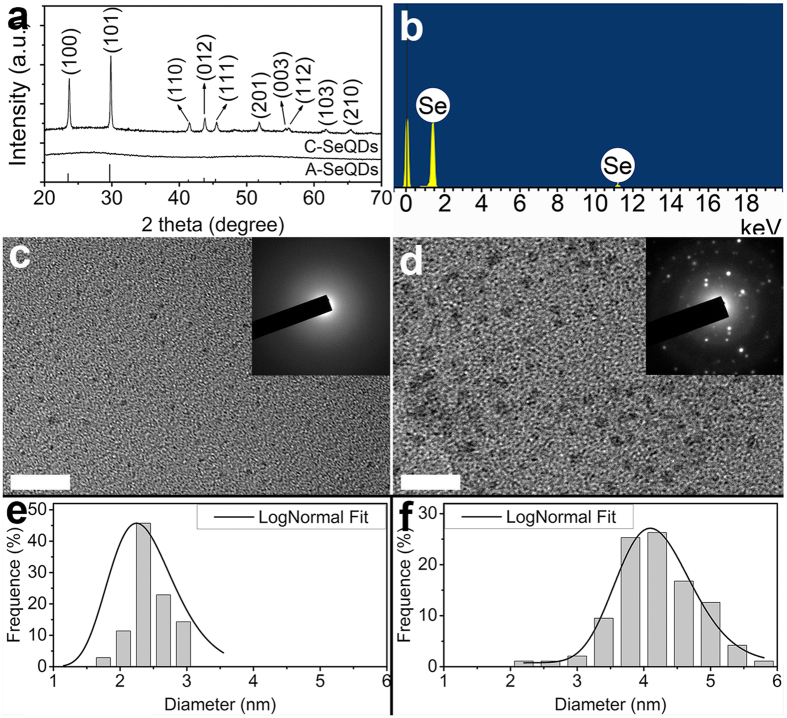
(**a**) XRD patterns of A-SeQDs (lower curve) and C-SeQDs (upper curve). (**b**) EDX spectrum of A-SeQDs. HRTEM images of (**c**) A-SeQDs and (**e**) C-SeQDs. Scale bar: 20 nm. Inset: SAED patterns. Size distribution analysis of (**d**) A-SeQDs and (**f**) C-SeQDs. The size distribution analysis through a lognormal distribution function from 100 quantum dots in an arbitrarily chosen area results in the narrow size distribution.

**Table 1:** List of the differentially expressed proteins and the quantitative changes after SeQDs treatment.

Protein name	Accession No.	Cellular function	Fold changesa	
			A-SeQD	C-SeQD
Stress response				
Thioredoxin reductase 1	Q16881_TRXR1	Oxidoreductase	2.994	1.335
Heat shock 70 kDa protein 1A	P08107_HSP71	Stress response	0.2542	0.7892
Heat shock protein beta-1	P04792_HSPB1	Stress response	0.0741	0.5671
Heat shock factor-binding protein 1-like protein 1	C9JCN9_HSBPL	Stress response	2.095	1.114
78 kDa glucose-regulated protein	P11021_GRP78	Endoplasmic reticulum stress process	5.382	2.234
Quinone oxidoreductase-like protein 1	O95825_QORL1	Oxidoreductase	1.616	1.0289
Annexin A4	P09525_ANXA4	Stress response	0.4103	0.6749
Annexin A2	P07355_ANXA2	Stress response	0.5883	0.905
Activator of 90 kDa heat shock protein ATPase homolog 1	O95433_AHSA1	Stress response	1.777	1.0832
Heat shock protein HSP 90-beta	P08238_HS90B	Stress response	0.659	
Endoplasmic reticulum resident protein 29	P30040_ERP29	Endoplasmic reticulum stress process	6.354	7.422
Signal pathway and transduction				
Protein Wnt-7a	O00755_WNT7A	Signal pathway	0.1193	0.5286
Regulator of G-protein signaling 3	P49796_RGS3	Signal pathway	0.1279	1.038
Rho GDP-dissociation inhibitor 1	P52565_GDIR1	Signal transduction	0.6514	0.3019
T-cell-specific surface glycoprotein CD28	P10747_CD28	Signal pathway	2.299	1.977
Glycoprotein hormone beta-5	Q86YW7_GPHB5	Signal pathway	0.1876	0.9304
14-3-3 protein sigma	P31947_1433S	Signal transduction	0.141	1.008
Protein biosynthesis and metabolism				
Eukaryotic translation initiation factor 3	O15371_EIF3D	Protein biosynthesis	0.1859	0.7888
Glycyl-tRNA synthetase	P41250_SYG	Protein biosynthesis	5.73	1.313
Ubiquitin carboxyl-terminal hydrolase isozyme L5	Q9Y5K5_UCHL5	Transcription regulation	15.37	1.403
T-complex protein 1 subunit alpha	P17987_TCPA	Protein folding	1.653	1.266
Reticulocalbin-1	Q15293_RCN1	Endoplasmic reticulum stress process	0.2422	0.6512
39S ribosomal protein L17	Q9NRX2_RM17	Protein translation	3.724	1.036
39S ribosomal protein L38	Q96DV4_RM38	Protein translation	1.599	
Sorting and assembly machinery component 50 homolog	Q9Y512_SAM50	Protein metabolism	0.6034	1.055
Diphthine methyl ester synthase	Q9H2P9_DPH5	Protein metabolic process	14.65	1.382
Elongation factor 2	P13639_EF2	Protein biosynthesis	0.6907	0.185
Tyrosine-protein phosphatase non-receptor type 4	P29074_PTN4	Protein metabolism	2.142	0.8319
T-complex protein 1 subunit theta	P50990_TCPQ	Proteins folding	0.6905	0.9746
Ras-related protein Rab-3D	O95716_RAB3D	Protein transport	0.6783	
RILP-like protein 2	Q969×0_RIPL2	Protein transport	1.6921	0.2279
Mitochondrial 2-oxodicarboxylate carrier	Q9BQT8_ODC	Protein transport	0.452	0.2889
Cell cycle				
Histone-binding protein	Q09028_RBBP4	Cell cycle regulation	0.6532	0.2111
Proliferating cell nuclear antigen	P12004_PCNA	DNA repair	0.0569	1.0010
tRNA (cytosine(34)-C(5))-methyltransferase	Q08J23_NSUN2	Cell division	0.1782	0.6879
E3 SUMO-protein ligase NSE2	Q96MF7_NSE2	Cell division	1.895	0.6007
Proteasome subunit alpha type-2	P25787_PSA2	Mitotic cell cycle	3.287	7.424
Mitotic spindle assembly checkpoint protein MDA1	Q9Y6D9_MD1L1	Cell division	2.675	0.9113
Cell adhesion and migration				
Moesin	P26038_MOES	Cell adhesion	0.629	1.035
Flotillin-1	O75955_FLOT1	Cell scaffolding protein	3.134	0.8988
Myosin-13	Q9UKX3_MYH13	Cell adhesion and migration	0.8122	0.5214
Ezrin	P15311_EZRI	Cell adhesion	0.593	0.9917
Angiopoietin-related protein 2	Q9UKU9_ANGL2	Cell autocrine	0.327	0.6654
Metabolism				
Delta(3,5)-Delta(2,4)-dienoyl-CoA isomerase	Q13011_ECH1	Lipid metabolism	0.2481	0.7873
Enoyl-CoA hydratase	P30084_ECHM	Lipid metabolism	0.1501	1.017
Thiosulfate sulfurtransferase	Q5T7W7_TSTD2	Small molecule metabolism	2.362	1.053
S-formylglutathione hydrolase	P10768_ESTD	Cellular metabolic process	2.76	1.809
Oxysterol-binding protein 2	Q969R2_OSBP2	Lipid Transport	1.902	0.7726
D-3-phosphoglycerate dehydrogenase	O43175_SERA	Glycolysis	5.79	1.546
Mitochondrial-processing peptidase subunit beta	O75439_MPPB	Cellular metabolic process	0.1289	0.9633
Methylthioribose-1-phosphate isomerase	Q9BV20_MTNA	Small molecule metabolic process	0.326	1.0174
V-type proton ATPase subunit B(Endomembrane)	P21281_VATB2	Energy metabolism	1.492	1.015
UDP-glucose 6-dehydrogenase	O60701_UGDH	Small molecule metabolism	0.6014	1.461
Bifunctional coenzyme A synthase	Q13057_COASY	Lipid metabolism	1.343	0.6184
Oxidation-reduction process				
Protein disulfide-isomerase A3	P30101_PDIA3	Cell redox homeostasis	4.967	1.349
Glyoxalase domain-containing protein 4	Q9HC38_GLOD4	Isomerase	1.855	1.073
Glutathione S-transferase omega-1	P78417_GSTO1	Oxidoreductase	1.669	1.536
Microtubule-associated protein tau	P10636_TAU	Microtubule assembly	1.5862	1.488
Echinoderm microtubule-associated protein-like 2	O95834_EMAL2	Microtubule assembly	0.6186	0.8546
Ribose-phosphate pyrophosphokinase 2	P11908_PRPS2	Nucleotide biosynthesis	3.255	0.575
Ribose-phosphate pyrophosphokinase 1	P60891_PRPS1	Nucleotide biosynthesis	1.586	3.208

^a^Protein spots were quantified based on the normalized average percentage of volume derived from ImageMaster 2D Platinum 7.0 software analysis. The data shown in the A-SeQDs and C-SeQDs column are the ratios of different protein expression levels in the treated cells to those in the control cells.
