# Supplementary material for: Mitochondria-Mediated Protein Regulation Mechanism of Polymorphs-Dependent Inhibition of Nanoselenium on Cancer Cells
Source: Sci Rep. 2016 Aug 12;6:31427. doi: 10.1038/srep31427 (PMC4981849; doi:10.1038/srep31427)
Supplement: Supplementary Information [file srep31427-s1.doc]

**Supplementary Information**

**Mitochondria-Mediated Protein Regulation Mechanism of Polymorphs-Dependent Inhibition of Nanoselenium on Cancer Cells**

Ge Wang^1,3 #^,Yuming Guo^1,2 #^, Gai Yang^1^, Lin Yang^1,2 *^, Xiaoming Ma^1^, Kui Wang^1^, Lin Zhu^1^, Jiaojiao Sun^1^, and Xiaobing Wang^1^, Hua Zhang^1^

1 Collaborative Innovation Center of Henan Province for Green Manufacturing of Fine Chemicals, Key Laboratory of Green Chemical Media and Reactions, Ministry of Education, Henan Normal University, Xinxiang, Henan 453007, P. R. China

2 Henan Key Laboratory of Green Chemical Media and Reactions, School of Chemistry and Chemical Engineering, Henan Normal University, Xinxiang, Henan 453007, P. R. China

3 School of Basic Medical Sciences, Xinxiang Medical University, Xinxiang, Henan 453003, P. R. China

**
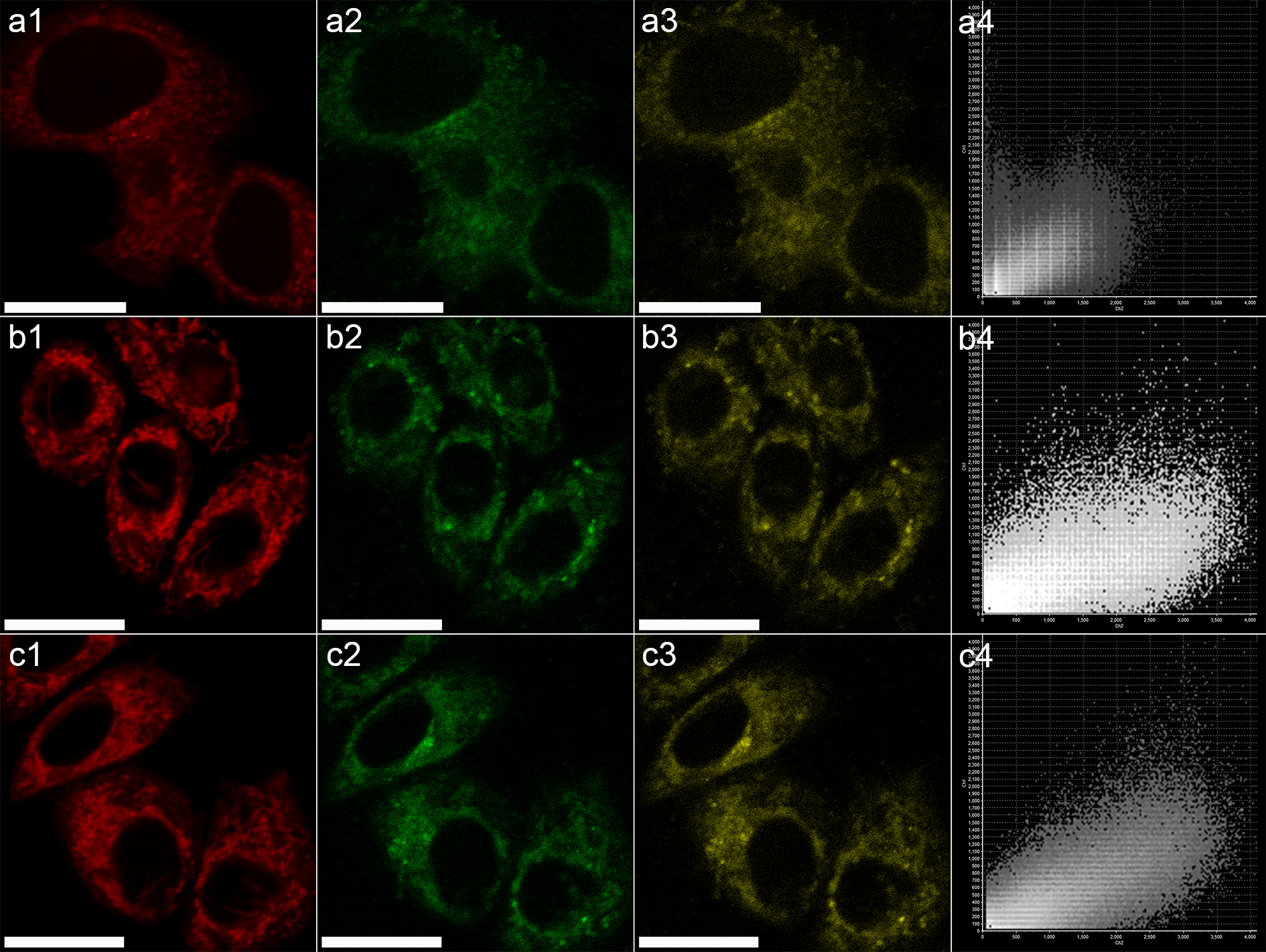
**

**Figure S1.** CLSM images of the different cells incubated with different samples. (a) MCF-7 cells incubated with C-SeQDs; (b) HeLa cells incubated with C-SeQDs; (c) BRL-3A cells incubated with C-SeQDs; (1) Mitochondria Tracker; (2) C-SeQDs; (3) Dark-field image of Mitochondria Tracker + C-SeQDs; (4) Intensity correlation plot of Mitochondria Tracker and C-SeQDs. Scale bar: 20 μm.

**
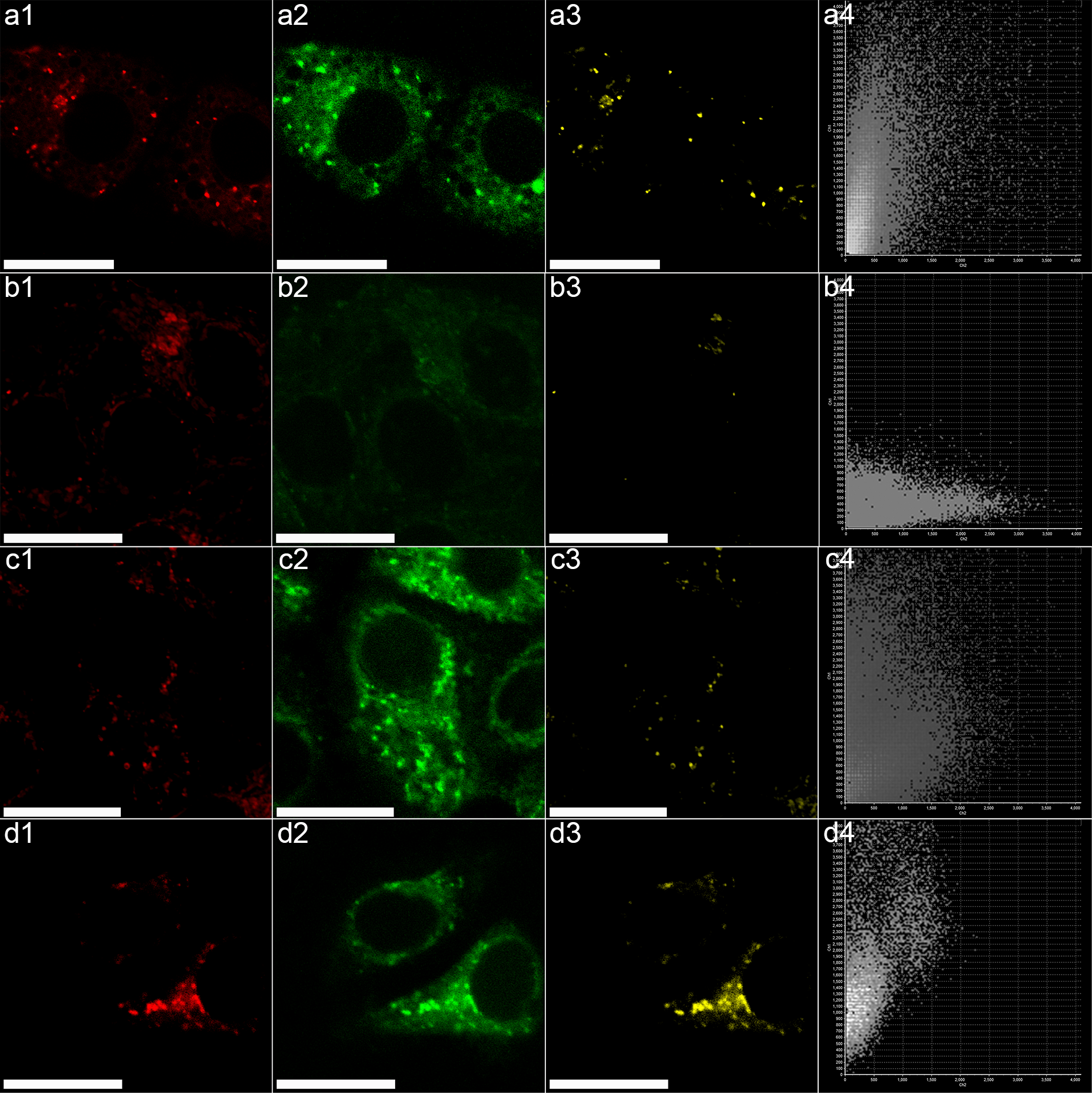
**

**Figure S2.** CLSM images of the different cells incubated with different samples. (a) Hep G2 cells incubated with A-SeQDs; (b) MCF-7 cells incubated with A-SeQDs; (c) HeLa cells incubated with A-SeQDs; (d) BRL-3A cells incubated with A-SeQDs; (1) Lysosome Tracker; (2) A-SeQDs; (3) Dark-field image of Lysosome Tracker + A-SeQDs; (4) Intensity correlation plot of Lysosome Tracker and A-SeQDs. Scale bar: 20 μm.


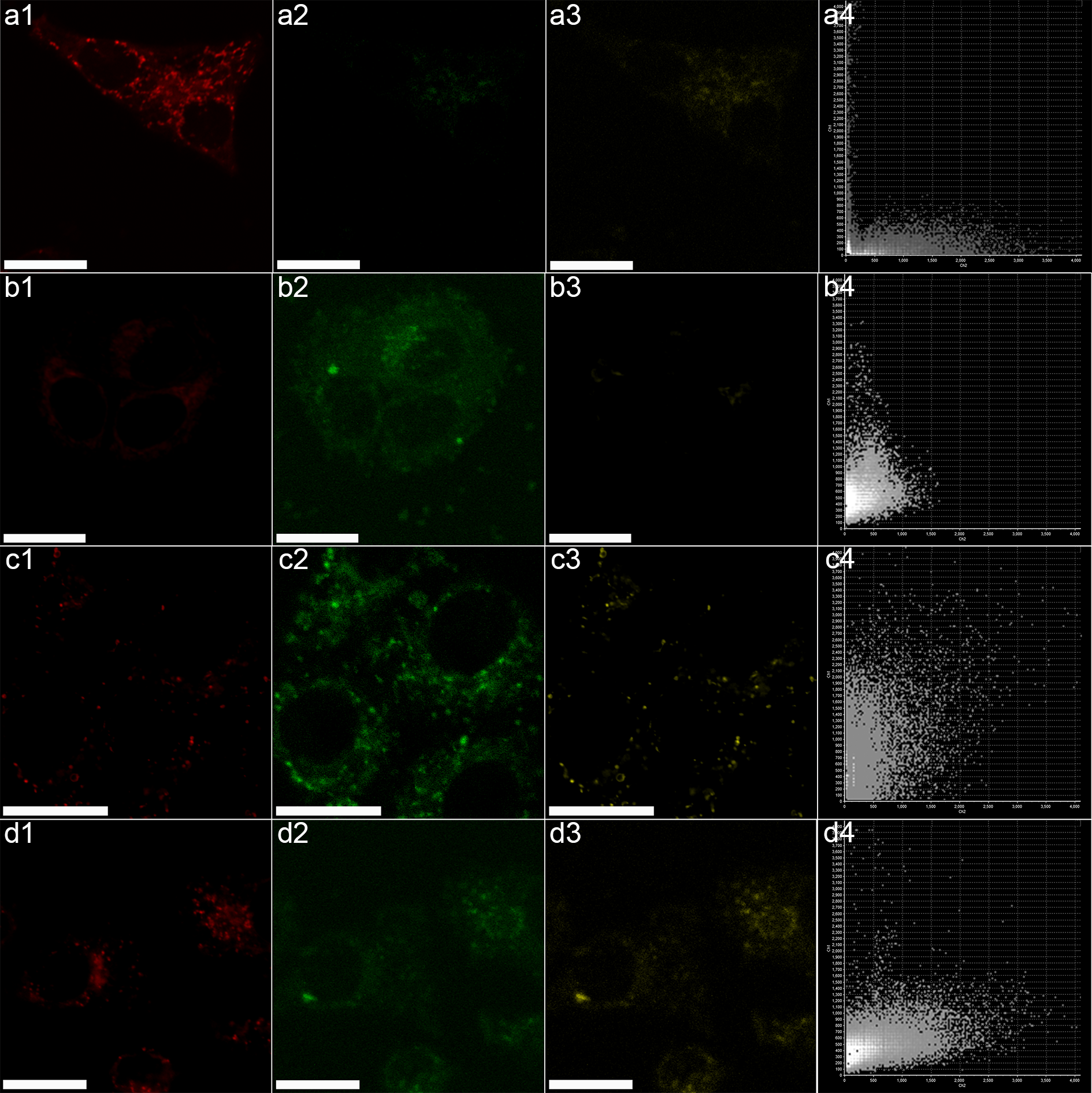


**Figure S3.** CLSM images of the different cells incubated with different samples. (a) Hep G2 cells incubated with C-SeQDs; (b) MCF-7 cells incubated with C-SeQDs; (c) HeLa cells incubated with C-SeQDs; (d) BRL-3A cells incubated with C-SeQDs; (1) Lysosome Tracker; (2) C-SeQDs; (3) Dark-field image of Lysosome Tracker + C-SeQDs; (4) Intensity correlation plot of Lysosome Tracker and C-SeQDs. Scale bar: 20 μm.

**
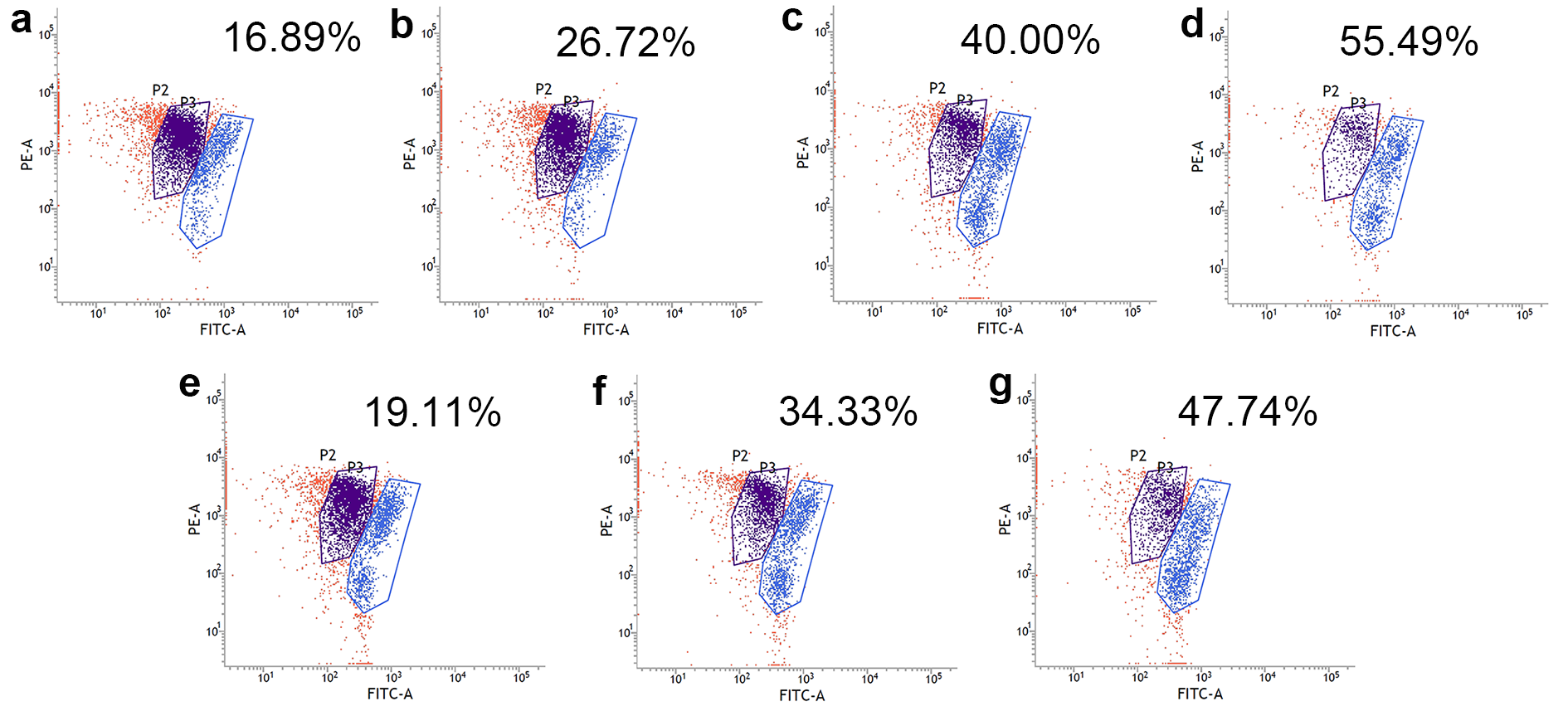
**

**Figure S4.** Loss of Δψm of Hep G2 cells after treated by different samples. (a) Control group; (b-d) Cells treated by 0.02, 0.06, and 0.16 mM A-SeQDs, respectively; (e-g) Cells treated by 0.02, 0.06, and 0.16 mM C-SeQDs, respectively. The data in each image represents the percentage of cells that losing Δψm.

In the current study, the status of mitochondria in Hep G2 cells exposed to different samples were examined and the results are shown in Fig. S4. In Fig. S4, the data in each image represents the percentage of cells that losing Δψm. From the data, the percentages of cells that losing Δψm after treated by different concentration of A-SeQDs increase gradually from 16.89 % of the control group (Fig. S4a) to 26.72 % for 0.02 mM (Fig. S4b), 40.00 % for 0.06 mM (Fig. S4c), and 55.49 % for 0.16 mM (Fig. S4d), respectively. Compared with A-SeQDs, the percentages of cells that losing Δψm after treated by different concentration of C-SeQDs increase gradually to 19.11% for 0.02 mM (Fig. S4e), 34.33% for 0.06 mM (Fig. S4f), and 47.74% for 0.16 mM (Fig. S4g), respectively. This shows that the two samples can both lead to the dose-dependent loss of Δψm, which will subsequently induce the apoptosis of cancer cells. More importantly, the effect of A-SeQDs on the loss of Δψm is obviously higher than that of C-SeQDs, correlated well with their apoptosis-inducing effects.

**Table S1 List of proteins identified by MALDI-TOF/TOF MS in Hep G2 cells.**

| **Accession No. ^a^** | **Protein**  **MW** | **Protein PI** | **Protein score / %**  **coverage^b^** | **Subcellular location** |
| --- | --- | --- | --- | --- |
| Q16881_TRXR1 | 71832 | 7.8 | 93/12 | Cytoplasm |
| P08107_HSP71 | 70294 | 5.4 | 87/17 | Cytoplasm |
| P04792_HSPB1 | 22782 | 5.98 | 52/9 | Cytoplasm |
| C9JCN9_HSBPL | 8378 | 4.7 | 54/14 | Cytoplasm |
| P11021_GRP78 | 72402 | 4.9 | 122/19 | Endoplasmic reticulum |
| O95825_QORL1 | 39071 | 5.4 | 82/17 | Cytoplasm |
| P09525_ANXA4 | 36088 | 5.8 | 78/24 | plasma membrane |
| P07355_ANXA2 | 38808 | 8.5 | 54/17 | extracellular matrix |
| O95433_AHSA1 | 38421 | 5.3 | 145/24 | Endoplasmic reticulum |
| P08238_HS90B | 83554 | 4.8 | 54/8 | Cytoplasm |
| P30040_ERP29 | 29032 | 7.5 | 57/12 | Endoplasmic reticulum |
| O00755_WNT7A | 71832 | 7.8 | 52/13 | extracellular matrix |
| P49796_RGS3 | 134134 | 5.9 | 51/5 | plasma membrane |
| P52565_GDIR1 | 23250 | 4.9 | 127/42 | Cytoplasm |
| P10747_CD28 | 25392 | 10.1 | 44/18 | plasma membrane |
| Q86YW7_GPHB5 | 14849 | 9.5 | 37/18 | extracellular |
| P31947_1433S | 27871 | 4.77 | 142/40 | Cytoplasm |
| O15371_EIF3D | 64560 | 5.7 | 60/14 | Cytoplasm |
| P41250_SYG | 83854 | 6.7 | 60/9 | Mitochondrion |
| Q9Y5K5_UCHL5 | 37868 | 5.1 | 55/17 | Cytoplasm, Nucleus |
| P17987_TCPA | 60819 | 5.7 | 91/13 | Cytoplasm |
| Q15293_RCN1 | 38866 | 4.7 | 55/16 | Endoplasmic reticulum |
| Q9NRX2_RM17 | 20095 | 10.6 | 52/32 | Mitochondrion |
| Q96DV4_RM38 | 71603 | 5.9 | 124/26 | Mitochondrion |
| Q9Y512_SAM50 | 52342 | 6.5 | 59/13 | Mitochondrion |
| Q9H2P9_DPH5 | 31973 | 5.1 | 50/20 | Cytoplasm |
| P13639_EF2 | 96246 | 6.4 | 93/12 | Cytoplasm, Nucleus |
| P29074_PTN4 | 106927 | 7.4 | 44/6 | plasma membrane |
| P50990_TCPQ | 60153 | 5.3 | 48/9 | Cytoplasm |
| O95716_RAB3D | 24480 | 4.6 | 60/22 | plasma membrane |
| Q969X0_RIPL2 | 24028 | 4.8 | 37/12 | Cytoplasm |
| Q9BQT8_ODC | 33453 | 10.0 | 39/16 | Mitochondrion |
| Q09028_RBBP4 | 47911 | 4.6 | 65/19 | Nucleus |
| P12004_PCNA | 29092 | 4.4 | 89/28 | Nucleus |
| Q08J23_NSUN2 | 87214 | 6.3 | 71/9 | Nucleus |
| Q96MF7_NSE2 | 28257 | 8.8 | 48/16 | Nucleus |
| P25787_PSA2 | 25996 | 7.7 | 87/26 | Cytoplasm, Nucleus |
| Q9Y6D9_MD1L1 | 83301 | 5.6 | 74/14 | Nucleus |
| P26038_MOES | 67892 | 6.0 | 90/17 | Cell membrane |
| O75955_FLOT1 | 47554 | 7.8 | 39/10 | Cell membrane |
| Q9UKX3_MYH13 | 224605 | 5.4 | 39/4 | Cytoplasm |
| P15311_EZRI | 69484 | 5.9 | 44/5 | Cell membrane |
| Q9UKU9_ANGL2 | 57582 | 7.8 | 34/6 | Extracellular |
| Q13011_ECH1 | 36136 | 9.2 | 107/28 | Mitochondrion |
| P30084_ECHM | 31823 | 9.4 | 57/20 | Mitochondrion |
| Q5T7W7_TSTD2 | 59764 | 7.1 | 43/11 | Cytoplasm |
| P10768_ESTD | 31956 | 6.6 | 63/25 | Cytoplasm |
| Q969R2_OSBP2 | 102172 | 6.1 | 42/6 | Cell membrane |
| O43175_SERA | 56651 | 6.3 | 66/20 | Cytoplasm |
| O75439_MPPB | 55073 | 6.4 | 61/12 | Mitochondrion |
| Q9BV20_MTNA | 39467 | 5.9 | 85/26 | Nucleus |
| P21281_VATB2 | 56807 | 5.5 | 98/18 | Cell membrane |
| O60701_UGDH | 55674 | 6.9 | 83/15 | Cytoplasm |
| Q13057_COASY | 62632 | 6.5 | 67/12 | Mitochondrion |
| P30101_PDIA3 | 57146 | 5.9 | 52/9 | Endoplasmic reticulum |
| Q9HC38_GLOD4 | 35170 | 5.3 | 75/27 | Mitochondrion |
| P78417_GSTO1 | 27833 | 6.3 | 64/21 | Cytoplasm |
| P10636_TAU | 79058 | 6.3 | 49/8 | Cell membrane |
| O95834_EMAL2 | 71603 | 5.9 | 143/22 | Cytoplasm |
| P11908_PRPS2 | 35146 | 6.2 | 42/15 | Nucleus |
| P60891_PRPS1 | 35325 | 6.6 | 115/26 | Nucleus |

^a^ Protein name and accession numbers were derived from Uniprot.

^b^ Protein score, percentage of coverage and Mr (Da)/PI were derived from MASCOT.

**Table S2 List of proteins related to ingenuity canonical pathways.**

| **Ingenuity Canonical Pathways** | **Molecules** |
| --- | --- |
| p70S6K Signaling | PDIA3,EEF2,MAPT,SFN |
| RhoGDI Signaling | EZR,ARHGDIA,MSN |
| Diphthamide Biosynthesis | EEF2,DPH5 |
| Aldosterone Signaling in Epithelial Cells | HSP90AB1,PDIA3,HSPA5,HSPB1 |
| p38 MAPK Signaling | MAPT,HSPB1 |
| Protein Ubiquitination Pathway | HSP90AB1,UCHL5,PSMA2,HSPA5,HSPB1 |
| Aryl Hydrocarbon Receptor Signaling | HSP90AB1,GSTO1,HSPB1 |
| NRF2-mediated Oxidative Stress Response | ERP29,TXNRD1,GSTO1 |
| Xenobiotic Metabolism Signaling | HSP90AB1,GSTO1,ESD |
| PRPP Biosynthesis | PRPS2,PRPS1 |
| Formaldehyde Oxidation II | ESD |
| Arsenate Detoxification I (Glutaredoxin) | GSTO1 |
| Superpathway of Serine and Glycine Biosynthesis I | PHGDH |
| PCP pathway | WNT7A,HSPB1 |
| DNA Methylation and Transcriptional Repression Signaling | RBBP4 |
| Endoplasmic Reticulum Stress Pathway | HSPA5 |
| Glutathione-mediated Detoxification | GSTO1 |
| GADD45 Signaling | PCNA |
| DNA damage-induced 14-3-3σ Signaling | SFN |
| Fatty Acid β-oxidation I | ECHS1 |
| ERK5 Signaling | SFN |
| Glutathione Redox Reactions II | PDIA3 |
| Lipid Antigen Presentation by CD1 | PDIA3 |
| Caveolar-mediated Endocytosis Signaling | FLOT1 |
| Phagosome formation | PDIA3 |
| Thioredoxin Pathway | TXNRD1 |
| Antigen Presentation Pathway | PDIA3 |
| Hypoxia Signaling in the Cardiovascular System | HSP90AB1 |
| Cell Cycle: G2/M DNA Damage Checkpoint Regulation | SFN |
| Myc Mediated Apoptosis Signaling | SFN |
| Regulation of Cellular Mechanics by Calpain Protease | EZR |
| Gap Junction Signaling | PDIA3 |
| Mismatch Repair in Eukaryotes | PCNA |
| tRNA Charging | GARS |
| UDP-D-xylose and UDP-D-glucuronate Biosynthesis | UGDH |
| BER pathway | PCNA |
| PPARα/RXRα Activation | HSP90AB1,PDIA3 |
| Mitotic Roles of Polo-Like Kinase | HSP90AB1 |
| Adenine and Adenosine Salvage VI | ADK |
| p53 Signaling | PCNA,SFN |
| LXR/RXR Activation | ECHS1 |
| Role of CHK Proteins in Cell Cycle Checkpoint Control | PCNA |
| Wnt pathway | PDIA3 |
| 14-3-3-mediated Signaling | PDIA3,MAPT,SFN |
| mTOR Signaling | EIF3D |
| Ephrin Receptor Signaling | RGS3 |
| Prostate Cancer Signaling | HSP90AB1 |
| UVA-Induced MAPK Signaling | PDIA3 |
| PPAR Signaling | HSP90AB1 |
| Telomerase Signaling | HSP90AB1 |
| CDK5 Signaling | MAPT |
| Signaling by Rho Family GTPases | EZR,MSN |
| HIPPO signaling | SFN |
| Protein Kinase A Signaling | PTPN4,PDIA3,SFN |
| P2Y Purigenic Receptor Signaling Pathway | PDIA3 |
| RhoA Signaling | EZR,MSN |
| VEGF Signaling | SFN |
| Synaptic Long Term Potentiation | PDIA3 |
| PI3K/AKT Signaling | HSP90AB1,SFN |
| Regulation of eIF4 and p70S6K Signaling | EIF3D |
| EIF2 Signaling | EIF3D |

This material is available free of charge via the Internet at [http://pubs.acs.org](http://pubs.acs.org/).

**
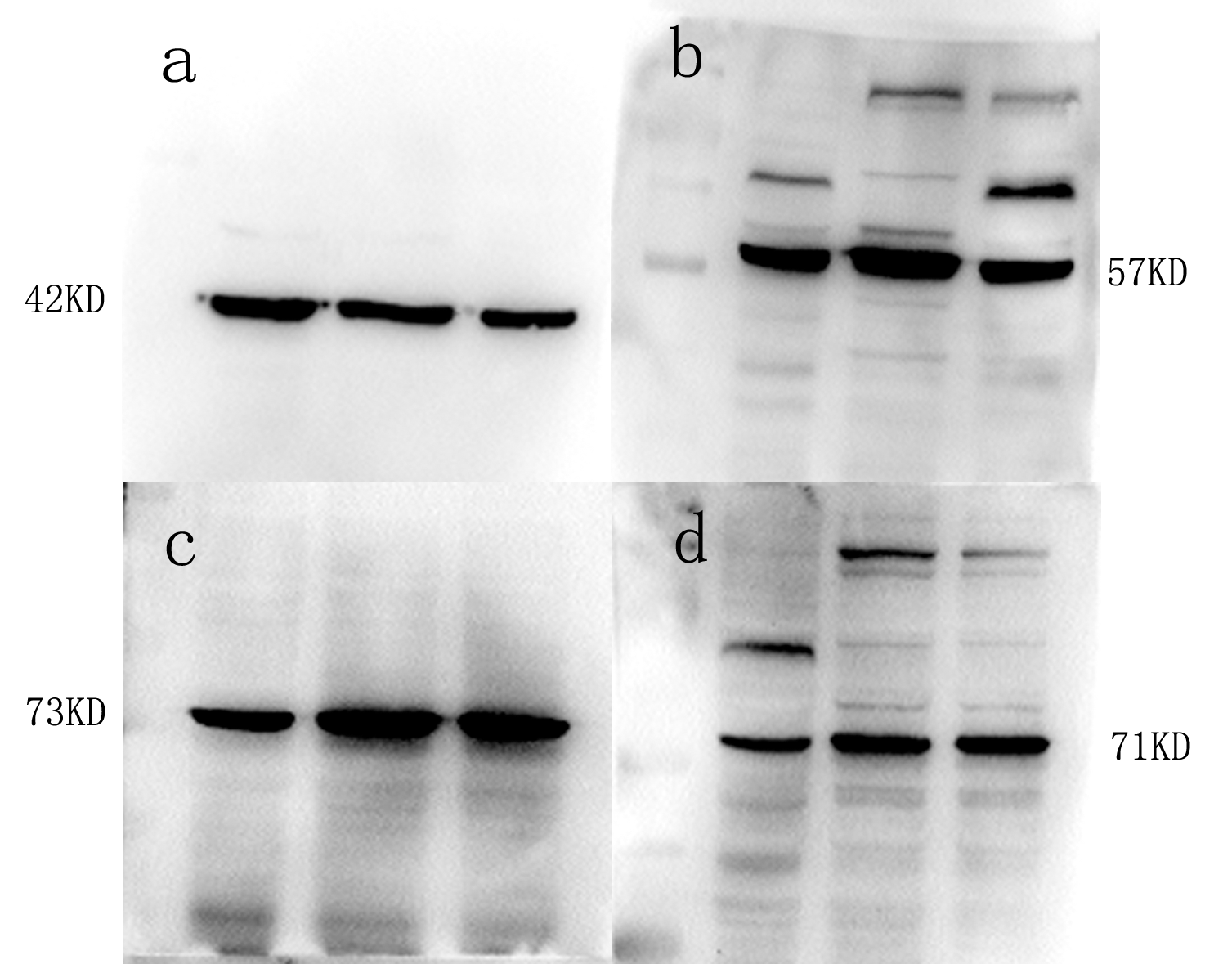
**

Figure S5. Effects of SeQDs on expression levels of β-actin, GRP78, PDIA3, and TRXR1 analyzed by Western blotting. (a) β-actin (b) PDIA3 (c) GRP78 (d) TRXR1. The full-length blots are shown.
